# "Is Cybermedicine Killing You?" - Codes of Ethics for Journalists

**DOI:** 10.2196/jmir.7.4.e45

**Published:** 2005-07-28

**Authors:** David Finer

As a medical journalist, as well as a public health/media researcher, I was intrigued by your recent articles [1,2] on the Cochrane debacle. Specifically, I am writing to complement the information in your article regarding retractions. You highlight a central but tricky part of the media-research universe. One of the articles mentioned in the editorial by Eysenbach and Kummervold was written by the extremely respected Swedish medical writer Inger Atterstam and was published in the conservative Stockholm daily Svenska Dagbladet on October 18, 2004. There was, however, a retraction of this article published on December 9, 2004, headlined "Researchers Retract Overview."

Your thesis still stands of course. The retraction article was smaller and less prominently placed than the original. Indeed, as you point out, most media did not publish any retraction at all.

The policy implications are important, though controversial. They relate to the larger issue of to what extent laws, formal or informal guidelines, or rules of thumb — implemented with due regard for the freedom of the press — can ameliorate the quality of reporting in general, and health reporting in particular.

In Sweden, media are, in practice, bound by a Code of Ethics for Press, Radio, and Television within a system of self-regulation involving four stakeholders, stipulating that media should "be generous with corrections...where relevant and to publish these...in suitable form and without delay...in such a way that they may reach the receivers of the original information" [3]. While this of course does not guarantee that retractions are given equal column space or airtime as the original news story, as the JMIR editorial suggests, it does demand that a correction be published.

One item in the recently published "A Statement of Principles for Health Care Journalists" by the Association of Health Care Journalists (AHCJ) in the United States addresses a related situation. It reads: "Consider public interest the primary criterion when choosing which stories to report. Follow up on those stories that serve a wider public interest. In particular, follow-up stories on subsequent failures, negative findings, or other reversals of fortune for investigational drugs, devices, or procedures should receive coverage comparable to that given initial positive reports" [4].

The Cochrane eHealth case highlights just one of many media inadequacies. In the interests of improved and more responsible journalism, not least in the health field, and with due respect for freedom of the press, there is a strong case to be made for bringing stronger pressure to bear on the media. Media representatives need to be more self-reflective about how their institutions mal/function, media research must become more interdisciplinary, and the media need to be held more accountable by the community.

However, when respected researchers, scientific organizations, or agencies themselves disagree on an issue or — as in this case — make mistakes, it is a tall order to expect health journalists to be wiser. Fortunately, nevertheless, health reporters do, not infrequently, manage to "expose fact, fiction, and fraud" [5].
